# Association of blood-based glial fibrillary acidic protein level with depression and suicidal ideation following traumatic brain injury with Glasgow Coma Scale score 13 to 15: a TRACK-TBI study

**DOI:** 10.1093/braincomms/fcaf123

**Published:** 2025-03-25

**Authors:** Shawn R Eagle, Raquel C Gardner, Sonia Jain, Xiaoying Sun, Ava Puccio, David Brent, Lindsay D Nelson, Michael A McCrea, Joseph T Giacino, David O Okonkwo, John K Yue, Geoffrey T Manley, Murray B Stein, The TRACK-TBI Investigators, Ann-Christine Duhaime, Adam R Ferguson, Shankar Gopinath, Ramesh Grandhi, C Dirk Keene, Christine Mac Donald, Amy Markowitz, Randall Merchant, Pratik Mukherjee, Laura B Ngwenya, Claudia Robertson, Andrea Schneider, David Schnyer, Sabrina R Taylor, Kevin Wang, Ross Zafonte

**Affiliations:** Department of Neurological Surgery, University of Pittsburgh, Pittsburgh, PA 15261, USA; Department of Epidemiology, Sheba Medical Center, Ramat Gan 52621, Israel; Department of Family Medicine and Public Health, University of California San Diego, La Jolla, CA 92093, USA; Department of Family Medicine and Public Health, University of California San Diego, La Jolla, CA 92093, USA; Department of Neurological Surgery, University of Pittsburgh, Pittsburgh, PA 15261, USA; Department of Neurological Surgery, University of Pittsburgh, Pittsburgh, PA 15261, USA; Department of Neurosurgery, Medical College of Wisconsin, Milwaukee, WI 53226, USA; Department of Neurosurgery, Medical College of Wisconsin, Milwaukee, WI 53226, USA; Department of Physical Medicine and Rehabilitation, Harvard University, Cambridge, MA 02129, USA; Department of Neurological Surgery, University of Pittsburgh, Pittsburgh, PA 15261, USA; Department of Neurological Surgery, University of California San Francisco, San Francisco, CA 94143, USA; Department of Neurological Surgery, University of California San Francisco, San Francisco, CA 94143, USA; Department of Family Medicine and Public Health, University of California San Diego, La Jolla, CA 92093, USA

**Keywords:** traumatic brain injury, suicidal ideation, GFAP, blood biomarker

## Abstract

Blood-based glial fibrillary acidic protein (GFAP) level within 24 h of traumatic brain injury (TBI) has been inversely associated with post-traumatic stress disorder at 6 months in the Transforming Research and Clinical Knowledge in Traumatic Brain Injury (TRACK-TBI) study. We sought to assess the relationship between day-of-injury GFAP and cumulative prevalence (CI) of depression or suicidal ideation in the first year after injury among patients presenting with Glasgow Coma Scale 13–15 who participated in Transforming Research and Clinical Knowledge in Traumatic Brain Injury (*n* = 1511). Multivariable logistic regression models were used to assess the association of day-of-injury GFAP levels with year 1 CI of depression or suicidal ideation adjusting for age, sex, prior TBI, psychiatric history and acute intracranial trauma on head computed tomography (CT) scan. Subgroup analyses categorized into ‘high’ and ‘low’ risk for mental health problems based upon a history of psychiatric disorder or TBI. Overall, 20.4% reported depression and 11.3% reported suicidal ideation in the first year. Participants with depression had significantly lower GFAP compared with participants without depression overall (median = 149.9 pg/mL versus 306.9 pg/mL, *P* < 0.001) and CT-negative high risk and CT-negative low risk subgroups. Participants with suicidal ideation had lower GFAP in the overall sample (155.8 pg/mL versus 299.1 pg/mL, *P* = 0.001). We found an interaction between GFAP and CT status, reflecting an inverse association of GFAP with cumulative depression among CT− subjects (adjusted odds ratio = 0.84, 95% CI: 0.77–0.92), but not among CT+ subjects. Blood biomarkers may warrant future investigation as potential predictors of depression following TBI in patients without evidence of acute intracranial trauma on CT scan.

## Teaser

Eagle *et al*. report that peripheral glial fibrillary acidic protein was inversely associated with odds of depression (but not suicidal ideation) within the first year of recovery in a cohort of >1500 adults with traumatic brain injury and negative head computed tomography scan at emergency department presentation.

## Graphical Abstract

**Graphical Abstract:**
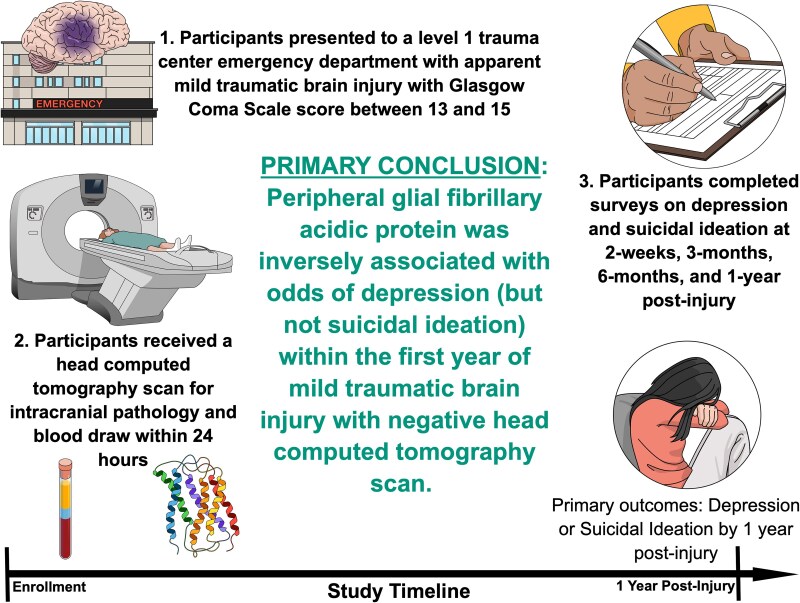

## Introduction

Traumatic brain injury (TBI) has been associated with increased risk for depression and suicidality, including a 1.6–4.1 times increased risk of suicide compared with the general population.^1-4^ A key limitation to this body of work is the lack of objective, clinically expedient biomarkers to identify those at highest risk for potential depression and suicidality who would benefit from more intensive mental health follow-up. Neuroimaging studies have shown that volume changes in dorsolateral pre-frontal cortex and caudate nucleus post-TBI, for example, correlate with higher rates of depression, anxiety, stress and worse neurocognition.^5-7^ However, neuroimaging is time- and resource-intensive and not always feasible within the clinical time-course of acute TBI management.

Glial fibrillary acidic protein (GFAP) and ubiquitin C-terminal hydrolase-L1 are two proteins, which have been approved by the U.S. FDA for the detection of intracranial injury following mTBI. GFAP has been shown to increase in peripheral blood following neurological trauma and decrease over time,^8^ but GFAP has also been linked to mental health disorders and suicide in populations both exposed and unexposed to head trauma.^9^ Kulbe *et al*.^10^ demonstrated in Transforming Research and Clinical Knowledge in Traumatic Brain Injury (TRACK-TBI) that lower GFAP concentrations on the day of mild TBI were associated with higher risk of developing post-traumatic stress disorder at 6 months post-injury. It is currently unknown if acute blood concentrations of GFAP are associated with post-TBI depression and/or suicidal ideation (SI) but identifying objective markers of depression and suicidality could improve clinical decision making for patients who may need long-term monitoring after TBI. The purpose of this study was to evaluate the association of day-of-injury (<24 h) GFAP with endorsing depression or SI throughout the first year of recovery from TBI with Glasgow Coma Scale (GCS) 13–15. Based upon the results of prior research,^11^ we hypothesized that mild TBI patients (GCS 13–15) with lower GFAP levels on the day of injury would have a higher risk of endorsing depression or SI in the first year. We also explored interactions between GFAP levels, head computed tomography (CT) findings and pre-injury depression/SI risk factors (e.g. history of psychiatric disorders or prior TBI) on the cumulative prevalence of depression and SI.

## Materials and methods

**Figure 1:**
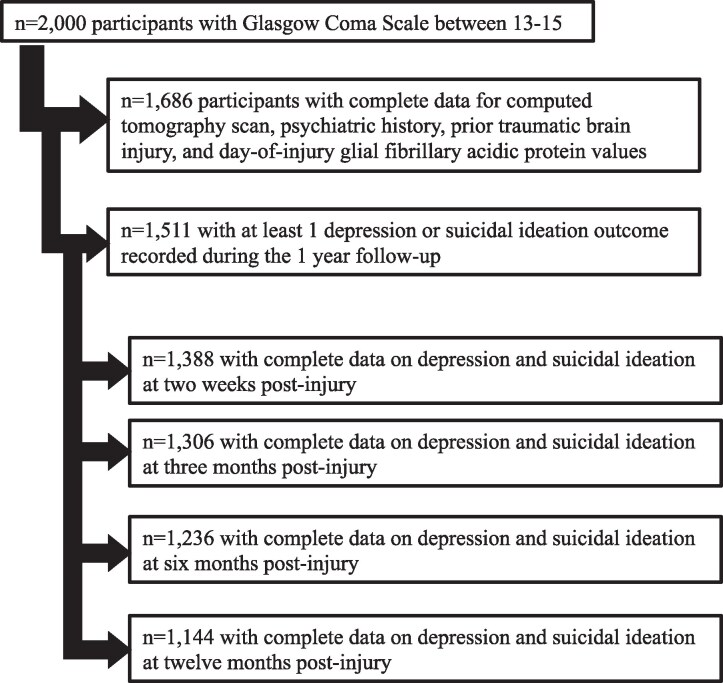
**CONSORT diagram.** Enroled and analysed subjects with traumatic brain injury and Glasgow Coma Scale score at presentation of 13–15.

This is an analysis of an existing database, including participants enroled in the TRACK-TBI study from 2014 to 2018. TRACK-TBI is a prospective, longitudinal, observational study of patients with TBI who presented to the emergency department of 18 level 1 trauma centres in the United States. Of 2000 (age ≥17) patients with TBI and GCS between 13 and 15 enrolled in TRACK-TBI, 1686 had complete data on computed tomography (CT) scan, medical history and plasma biospecimens from day-of-injury. Of those, 1511 had at least one outcome measure at the follow-up visits within the first year of injury and were included in analyses (see CONSORT diagram in Fig. 1). The included cohort was intentionally limited to GCS 13–15 at presentation as psychiatric sequelae disproportionately affect patients with ‘milder’ TBI compared with more severe TBI.^11^ Ethical approval for human subjects research was obtained from each of the 23 participating sites’ institutional review board. Participants provided written informed consent prior to initiating study procedures in accordance with the Declaration of Helsinki.

### Procedures

Participants or their legally authorized representatives provided written informed consent to participate after being approached by a member of the research team in the ED. Enroled patients provided blood samples within 24 h of injury. Participants were included in the study if presenting to the ED within 24 h of non-penetrating head injury who had head CT performed. Those with subsequently negative head CT were required to meet clinical criteria for TBI: traumatically induced amnesia, loss of consciousness, or alteration of mental status not better explained by a non-traumatic aetiology.^12^ Exclusion criteria included pregnancy, incarceration, penetrating TBI, nonsurvivable physical trauma in the opinion of the investigator in consult with the emergency medical team, debilitating mental health disorders or neurological disease (e.g. stroke, dementia, tumour), magnetic resonance imaging contraindications.

Participants completed a standardized outcome assessment battery at 2 weeks, 3 months, 6 months and 12 months post-injury which included self-reported demographics and medical history [i.e. psychiatric history (yes/no), prior TBI (yes/no)], the Brief-Symptom Inventory-18, the Patient Health Questionnaire-9. Baseline psychiatric history was defined as self-reporting any psychiatric diagnosis prior to the incident TBI (e.g. depression, anxiety). Follow up rates for 2 weeks (91.9%; *n* = 1388), 3 months (86.4%; *n* = 1306), 6 months (81.8%; *n* = 1236) and 12 months post-injury (75.7%; *n* = 1144) decreased slightly over time. The 2 week, 6- and 12-month follow ups were ideally conducted in person, or by phone if necessary, while the 3-month follow-up was always conducted by phone. Depression was defined as Patient Health Questionnaire-9 total> = 15 based upon prior research establishing the validity of this cut-off for moderate severity major depressive disorder.^13^ Reports of any level of suicidal thoughts on Patient Health Questionnaire-9 (item 9 rating >0) or BSI 18 (item 17 rating >0) triggered administration of the Columbia—Suicide Severity Rating Scale. SI was defined as a positive response to any of the 5 past-month questions in the Columbia—Suicide Severity Rating Scale SI section. The primary outcomes were cumulative prevalence of depression (i.e. endorsing depression over the cut-off score 1+ times in the first year) and cumulative prevalence of SI in the first year post-TBI (i.e. endorsing SI 1+ times in the first year). Participants who endorsed depression or SI multiple times within the first year were only counted once for multivariable modelling. Further, participants who endorsed depression or SI once but not at subsequent timepoints were still counted as positive for depression or SI.

### Blood biomarkers

Blood samples were collected within 24 h of injury. Samples were processed and stored according to the Traumatic Brain Injury Common Data Elements Biospecimens and Biomarkers Working Group consensus recommendations for plasma and serum preparation.^14^ The first batch of plasma GFAP concentrations was measured using the prototype point-of-care i-STAT Alinity System (Abbott Laboratories, Abbott Park, IL, USA), which had a limit of detection of 15 pg/mL, limit of quantitation of 25 pg/mL and coefficient of variation of 2.8–14.2%. The second batch of plasma GFAP concentrations were measured on the prototype core lab ARCHITECT platform (Abbott Laboratories, Abbott Park, IL, USA) for faster throughput. The ARCHITECT platform had a limit of detection of 2 pg/mL, limit of quantitation of 5 pg/mL and coefficient of variance of 2.0–5.6%. Upper limits for both platforms was 50 000 pg/mL. The two assays were highly correlated (*r* = 0.93–0.99), and ARCHITECT values were converted to iSTAT equivalents by use of two previously derived equations.^15^

### CT imaging

CT images were independently evaluated by a board-certified neuroradiologist according to the National Institutes of Neurological Disorders and Stroke Common Data Elements Neuroimaging Working Group recommendations.^16^ Images were deidentified prior to uploading to a central imaging database, and the neuroradiologist was masked to both the patient's identity and other clinical information. The CT scan was considered positive (CT+) if there was evidence of acute intracranial pathology related to TBI.

### Statistical analysis

Day-of-injury GFAP levels were compared between participants with and without depression/SI during the first year post-injury using the Wilcoxon Rank Sum test in the overall sample. Wilcoxon Rank Sum tests were also used to compare subgroups of CT+ versus CT−, pre-injury high risk for mental health (defined as having prior psychiatric history and/or prior TBI) versus low risk. The high risk variables were selected based upon available literature associating prior psychiatric history and TBI with subsequent psychiatric sequelae.^11,17-19^ Participants were stratified by CT status (positive versus negative) for comparison as day-of-injury GFAP is known to be meaningfully higher in patients with evidence for intracranial pathology.^20^ Multivariable logistic regression models were used to study the association of GFAP levels (in log scale) with cumulative prevalence of depression or SI.^11,21^ To assess the potential bias of the cumulative outcomes due to loss to follow-up, the multivariable logistic regression models were repeated including only participants who completed the 1-year follow-up visit for a sensitivity analysis. All multivariable models were adjusted for known risk factors of psychiatric sequelae including age, gender, CT status (CT+ versus CT−), prior TBI, and baseline psychiatric history. Adjusted odds ratios and 95% confidence intervals (95 CIs) were reported from the models. All analyses were conducted using R (version 4.1.2; www.r-project.org).

## Results

Demographics and baseline clinical characteristics of the study cohort were summarized in Table 1 by outcome status. Overall, 20.4% of the sample had depression and 11.3% had SI in the first year post-injury. Prevalence of depression at each timepoint was: 2 weeks (13.3%; *n* = 184), 3 months (9.7%; *n* = 126), 6 months (9.7%; *n* = 120) and 12 months (8.1%; *n* = 93). Prevalence of SI at each timepoint was: 2 weeks (4.5%; *n* = 63), 3 months (4.7%; *n* = 61, 6 months (5.4%; *n* = 67) and 12 months (5.2%; *n* = 59). Female participants and Black participants were more likely to have depression in the first year. Participants with depression and participants with SI in the first year post-injury were more likely to have a psychiatric history, prior TBI and negative head CT scan compared with participants without depression or SI.

**Table 1:** Demographic and clinical characteristics by outcomes

	Suicidal ideation (*n* = 171)	No suicidal ideation (*n* = 1340)	*P*	Depression (*n* = 308)	No depression (*n* = 1203)	*P*
Age	36.0 ± 14.2	41.3 ± 17.5	0.001*	39.6 ± 14.5	40.9 ± 17.9	0.81
Years of education	13.1 ± 2.3	13.7 ± 3.0	0.004*	12.9 ± 2.6	13.8 ± 3.0	<0.001*
Female sex	61 (35.7)	451 (33.7)	0.61	142 (46.1)	370 (30.8)	<0.001*
Race						
White	130 (76.5)	1031 (77.2)	0.29	219 (71.1)	942 (78.7)	<0.001*
Black	33 (19.4)	215 (16.1)		78 (25.3)	170 (14.2)	
Other	7 (4.1)	89 (6.7)		11 (3.6)	85 (7.1)	
Hispanic ethnicity	33 (19.3)	259 (19.4)	0.99	58 (18.8)	234 (19.5)	0.81
Mechanism						
Motor vehicle accident	99 (58.2)	771 (57.7)	0.91	171 (55.9)	699 (58.2)	0.75
Fall	47 (27.7)	353 (26.4)		83 (27.1)	317 (26.4)	
Violence/assault	9 (5.3)	91 (6.8)		24 (7.8)	76 (6.3)	
Other	15 (8.8)	122 (9.1)		28 (9.2)	109 (9.1)	
Psychiatric history	83 (48.5)	266 (19.9)	<0.001*	122 (39.6)	227 (18.9)	<0.001*
Depression	64 (37.4)	165 (12.3)	<0.001*	85 (27.6)	144 (12.0)	<0.001*
Anxiety	50 (29.2)	144 (10.8)	<0.001*	80 (26.0)	114 (9.5)	<0.001*
Bipolar disorder	10 (5.9)	14 (1.0)	<0.001*	11 (3.6)	13 (1.1)	<0.001*
Schizophrenia	4 (2.3)	1 (0.1)	<0.001*	2 (0.7)	3 (0.3)	<0.001*
Post-traumatic stress disorder	10 (5.9)	18 (1.3)	<0.001*	16 (5.2)	12 (1.0)	<0.001*
Other	15 (8.8)	24 (1.8)	<0.001*	14 (4.6)	25 (2.1)	<0.001*
Highest level of care			0.45			0.45
ED discharge	47 (27.5)	362 (27.0)		82 (26.6)	327 (27.2)	
Hospital admit no ICU	80 (46.8)	572 (42.7)		142 (46.1)	510 (42.4)	
Hospital admit with ICU	44 (25.7)	450 (29.8)		84 (27.3)	366 (30.4)	
Prior TBI	61 (35.7)	277 (20.7)	<0.001*	112 (36.4)	226 (18.8)	<0.001*
Glasgow Coma Scale score						
13	6 (3.5)	62 (4.6)	0.85	15 (4.9)	53 (4.4)	0.92
14	31 (18.1)	247 (18.4)		57 (18.5)	221 (18.4)	
15	134 (78.4)	1031 (76.9)		236 (76.6)	929 (77.2)	
Loss of Consciousness	146 (85.4)	1102 (82.4)	0.02*	259 (84.1)	989 (82.4)	0.16
Post-traumatic amnesia	133 (77.8)	1002 (74.9)	0.76	240 (77.9)	895 (74.5)	0.15
Positive computed tomography	47 (27.5)	495 (36.9)	0.02*	89 (28.9)	453 (37.7)	0.004*
Contusion	18 (10.5)	158 (11.8)	0.71	35 (11.4)	141 (11.7)	0.92
Subarachnoid haemorrhage	29 (17.0)	320 (23.9)	0.04*	58 (18.8)	291 (24.2)	0.05
Subdural haematoma	22 (12.9)	222 (16.6)	0.23	45 (14.6)	199 (16.6)	0.44
Epidural haematoma	9 (5.3)	72 (5.4)	>0.99	16 (5.2)	65 (5.4)	>0.99
Intraventricular haemorrhage	1 (0.6)	19 (1.4)	0.72	4 (1.3)	16 (1.3)	>0.99
Injury severity score total^a^	10.5 ± 7.8	12.4 ± 7.9	0.003*	10.9 ± 7.5	12.6 ± 8.0	0.005*
Injury severity score-non head^a^	4.3 ± 5.2	5.6 ± 6.7	0.05	5.1 ± 6.8	5.5 ± 6.5	0.13
Blood sampling time (hours)			0.65			0.35
0–4	15 (9.0)	103 (8.1)		17 (5.7)	101 (8.8)	
5–8	18 (10.8)	189 (14.8)		42 (14.2)	165 (14.4)	
9–12	21 (12.7)	160 (12.5)		38 (12.8)	143 (12.5)	
13–16	30 (18.1)	230 (18.0)		56 (18.9)	204 (17.8)	
17–20	42 (25.3)	255 (19.9)		65 (22.0)	232 (20.2)	
21–24	38 (22.9)	316 (24.7)		76 (25.7)	278 (24.2)	
>24	2 (1.2	26 (2.0)		2 (0.7)	26 (2.3)	

^*^Statistically significant at *P* < 0.05.

^a^Only collected in hospitalized participants.

Day-of-injury GFAP levels were significantly lower for participants with depression compared with those without depression [median (Q1, Q3) = 149.9 (22.7, 733.8) pg/mL versus 306.9 (85.1, 843.3), *P* < 0.001] in the overall sample and in subgroups with CT- high pre-injury risk (*P* < 0.001) and CT- low pre-injury risk (*P* = 0.036), but not in CT+ high or low pre-injury risk subgroups (see Table 2). This suggests an interaction between CT status and GFAP levels on the cumulative depression outcome.

**Table 2:** Depression subgroup comparisons of day of injury glial fibrillary acidic protein [GFAP (pg/mL); median (interquartile range)] stratified by participants with high risk for post-injury psychiatric symptoms and low risk for psychiatric symptoms

	High risk			Low risk		
	Depression	No depression	*P*	Depression	No depression	*P*
CT+	1132.0 (248.6, 2082.0)	707.9 (342.9, 1641.3)	0.631	871.5 (501.1, 1842.7)	809.6 (302.0, 2007.9)	0.316
CT-	40.0 (12.2, 210.4)	120.9 (25.0, 367.2)	<0.001*	105.2 (21.1, 382.1)	196.4 (52.2, 454.1)	0.036*
GCS15	85.8 (17.5, 354.7)	161.1 (39.0, 520.5)	0.013*	271.9 (30.1, 763.2)	261.9 (74.1, 673.4)	0.487
GCS13–14	241.6 (25.1, 1053.8)	602.3 (251.4, 1480.6)	0.009*	633.6 (128.8, 1601.7)	934.4 (334.3, 2177.8)	0.030*

High risk indicates a participant had psychiatric history and/or prior TBI and low risk indicates having neither.

^*^Denotes statistical significance at *P* < 0.05.

Day of injury GFAP levels were significantly lower for participants with SI compared with those without [155.8 (37.9, 551.7) pg/mL versus 299.1 (74.2, 840.4) pg/mL, *P* = 0.001] in the overall sample, but no significant difference was found in any of the subgroups (see Table 3).

**Table 3:** Suicidal ideation subgroup comparisons of day of injury GFAP [(pg/mL); median (interquartile range)] stratified by participants with high risk for post-injury psychiatric symptoms and low risk for psychiatric symptoms

	High risk			Low risk		
	Depression	No depression	*P*	Depression	No depression	*P*
CT+	686.4 (171.9, 1928.3)	825.3 (350.9, 1723.9)	0.359	763.5 (450.1, 1926.6)	833.8 (337.7, 1995.4)	0.799
CT-	70.3 (17.0, 190.8)	93.4 (19.4, 350.4)	0.205	131.0 (57.7, 375.3)	185.7 (42.6, 453.5)	0.592
GCS15	108.7 (19.7, 255.5)	148.8 (26.5, 514.3)	0.062	183.4 (76.9, 629.1)	266.5 (69.2, 690.1)	0.862
GCS13–14	428.5 (94.9, 1123.9)	526.1 (204.0, 1421.0)	0.518	543.0 (150.1, 934.4)	928.0 (318.7, 2153.2)	0.039*

High risk indicates a participant had psychiatric history and/or prior TBI and low risk indicates having neither.

^*^Denotes statistical significance at *P* < 0.05.

### Multivariable modelling for predicting depression and SI within the first year

Adjusting for age, sex, psychiatric history and prior TBI, we found a significant interaction effect between GFAP levels and CT status, indicating an inverse association of GFAP levels with depression in the first year post-injury among CT-subjects (adjusted odds ratios = 0.84, 95% CI: 0.77–0.92 per log unit increase; see Table 4).

**Table 4:** Logistic regression model for predicting cumulative depression within the first year post-traumatic brain injury with Glasgow Coma Scale score between 13 and 15 (*n* = 1511)

	Adj. OR	95% CI	*P*
Day-of-injury GFAP	0.89	0.82–0.97	0.006*
Positive computed tomography scan	1.00	0.72–1.39	1.00
Age	1.00	0.99–1.01	0.42
Female sex	1.76	1.34–2.32	<0.001*
Psychiatric history	2.27	1.71–3.02	<0.001*
Prior traumatic brain injury	2.32	1.74–3.09	<0.001*
Model including interaction of GFAP and computed tomography scan (*n* = 1511)			
Day-of-injury GFAP (in log scale)	0.84	0.77–0.92	<0.001*
Positive Computed Tomography Scan	0.83	0.56–1.21	0.33
Age	1.00	0.99–1.01	0.49
Female Sex	1.78	1.35–2.34	<0.001*
Psychiatric History	2.23	1.68–2.97	<0.001*
Prior Traumatic Brain Injury	2.30	1.72–3.07	<0.001*
Day-of-injury GFAP (in log scale) × Positive Computed Tomography Scan	1.30	1.06–1.59	0.01*
Restricted to participants with negative computed tomography scans (*n* = 969)			
Day-of-injury GFAP >35 pg/mL	0.51	0.36–0.71	<0.001*
Age	1.00	0.99–1.01	0.65
Female Sex	1.90	1.36–2.65	<0.001*
Psychiatric History	2.29	1.62–3.22	<0.001*
Prior Traumatic Brain Injury	2.31	1.63–3.27	<0.001*
Restricted to participants who completed the 1-year post-injury visit (*n* = 1144)			
Day-of-injury GFAP	0.85	0.70–0.87	0.001*
Positive computed tomography scan	1.00	0.50–1.20	0.98
Age	1.00	0.99–1.01	0.43
Female sex	1.61	1.18–2.19	0.003*
Psychiatric history	2.64	1.92–3.64	<0.001*
Prior traumatic brain injury	2.45	1.76–3.42	<0.001*
Restricted to participants who completed the 1-year post-injury visit and including an interaction term between GFAP and computed tomography scan (*n* = 1144)			
Day-of-injury GFAP	0.78	0.70–0.87	<0.001*
Positive computed tomography scan	0.78	0.50–1.20	0.25
Age	1.00	0.99–1.01	0.52
Female sex	1.63	1.20–2.23	0.002*
Psychiatric history	2.58	1.87–3.57	<0.001*
Prior traumatic brain injury	2.43	1.74–3.39	<0.001*
Day-of-injury GFAP × positive computed tomography scan	1.44	1.14–1.80	0.002*
Restricted to participants who completed the 1-year post-injury visit and had a negative computed tomography scan (*n* = 725)			
Day-of-injury GFAP >35 pg/mL	0.43	0.29–0.64	<0.001*
Age	1.00	1.00–1.01	0.88
Female sex	1.68	1.15–2.44	0.007*
Psychiatric history	2.82	1.91–4.15	<0.001*
Prior traumatic brain injury	2.45	1.64–3.67	<0.001*

^*^Statistically significant at *P* < 0.05.

Sensitivity models including only those participants who completed the 1-year follow up visit had consistent results with above (Table 4).

The multivariable model for SI showed no significant association between day-of-injury GFAP levels and cumulative prevalence of SI after adjusting for covariates (see Table 5).

**Table 5:** Logistic regression model for predicting cumulative suicidal ideation within the first year post-traumatic brain injury with Glasgow Coma Scale score between 13 and 15 (*n* = 1511)

	Adj. OR	95% CI	*P*
Day-of-injury GFAP (in log scale)	0.96	0.87–1.07	0.44
Positive computed tomography scan	0.91	0.59–1.39	0.65
Age	0.98	0.97–0.99	<0.001*
Female sex	0.86	0.60–1.23	0.40
Psychiatric history	3.83	2.70–5.42	<0.001*
Prior traumatic brain injury	1.76	1.23–2.51	0.002*
Model including interaction of GFAP and computed tomography scan (*n* = 1511)			
Day-of-injury GFAP	0.96	0.86–1.08	0.52
Positive computed tomography scan	0.92	0.57–1.47	0.73
Age	0.98	0.97–0.99	<0.001*
Female sex	0.86	0.60–1.23	0.40
Psychiatric history	3.83	2.71–5.43	<0.001*
Prior traumatic brain injury	1.76	1.23–2.51	0.002*
Day-of-injury GFAP × positive computed tomography scan	0.98	0.76–1.27	0.89
Restricted to participants with negative computed tomography scans (*n* = 969)			
Day-of-injury GFAP >35 pg/mL	1.06	0.68–1.65	0.81
Age	0.98	0.97–1.00	0.007*
Female sex	0.91	0.60–1.38	0.65
Psychiatric history	4.12	2.72–6.23	<0.001*
Prior traumatic brain injury	1.79	1.18–2.72	0.006*
Restricted to participants who completed the 1-year post-injury visit (*n* = 1144)			
Day-of-injury GFAP	0.93	0.83–1.05	0.25
Positive computed tomography scan	1.09	0.68–1.75	0.73
Age	0.98	0.96–0.99	<0.001*
Female sex	0.80	0.53–1.19	0.27
Psychiatric history	4.10	2.77–6.07	<0.001*
Prior traumatic brain injury	1.96	1.31–2.95	0.001*
Restricted to participants who completed the 1-year post-injury visit and including an interaction term between GFAP and computed tomography scan (*n* = 1144)			
Day-of-injury GFAP	0.91	0.79–1.04	0.17
Positive computed tomography scan	1.01	0.59–1.71	0.98
Age	0.98	0.97–0.99	<0.001*
Female sex	0.80	0.53–1.19	0.27
Psychiatric history	4.07	0.28–6.03	<0.001*
Prior traumatic brain injury	1.95	1.30–2.93	0.001*
Day-of-injury GFAP × positive computed tomography scan	1.11	0.84–1.47	0.47
Restricted to participants who completed the 1-year post-injury visit and had a negative computed tomography scan (*n* = 725)			
Day-of-injury GFAP >35 pg/mL	0.86	0.52–1.43	0.56
Age	0.97	0.96–0.99	0.002*
Female sex	0.89	0.55–1.44	0.63
Psychiatric history	4.46	2.77–7.17	<0.001*
Prior traumatic brain injury	2.08	1.28–3.38	0.003*

^*^Statistically significant at *P* < 0.05.

## Discussion

In this analysis of a prospective cohort study of >1500 participants with a ‘mild’ TBI (i.e. presenting GCS of 13–15), lower peripheral GFAP within 24 h of injury was associated with meeting a clinical cut-off for depression within the first year of recovery. No such association was seen for SI. Subgroup analyses revealed lower GFAP levels in patients with negative presenting head CT scans who met criteria for depression in the first year post-injury, regardless of pre-injury risk factors. This study contributes to the literature by demonstrating that blood biomarkers of acute brain injury may be negatively associated with mental health sequelae such as depression in patients with TBI and negative initial head CT scan, but future work is necessary to better understand this association.^22^ Clinicians who have access to day-of-injury peripheral GFAP levels (e.g. emergency department providers) should be aware that lower GFAP does not necessarily mean there will not be long-term psychiatric sequelae from TBI.

One possible hypothesis for our findings may be that depression is associated with lower baseline (i.e. pre-injury) GFAP concentrations, resulting in lower post-injury GFAP levels after TBI in comparison to TBI participants without pre-injury depression. Evidence suggests that glial dysfunction may be a mechanism contributing to liability for a major depressive disorder.^23-25^ Prior work on post-mortem brain tissue from participants with depression has reported decreased numbers, density and protein expression of glial cells in the hippocampus and cortico-limbic regions.^24^ Hviid *et al*.^23^ previously reported that depressed patients had ∼17% lower GFAP serum concentrations at baseline compared with healthy controls. Nagy *et al*.^26^ reported a downregulation of GFAP transcription and protein levels in the mediodorsal thalamus and caudate nucleus on autopsy in depressed participants who committed suicide compared with healthy controls. Studies on brain tissue of depressed participants have shown decreased GFAP immunoreactivity and less dense GFAP-labelled astrocytes compared with matched healthy controls.^27^ Similarly, in our prior study of TRACK-TBI patients with mTBI, patients with post-traumatic stress disorder at 6 months had lower day-of-injury plasma GFAP concentrations than patients without post-traumatic stress disorder.^10^ Prior work has identified lower GFAP expression in hippocampi of rat models of post-traumatic stress disorder.^28,29^ It should be noted that this potential hypothesis requires additional exploration, as it is unknown if patients who reported pre-injury psychiatric history had a formal diagnosis and/or were treated.

Another hypothesis could be that some participants with lower acute GFAP may not have suffered a TBI, despite their reporting of a traumatic mechanism of injury and at least 1 traumatically-induced clinical sign of TBI. The lack of objective assessments for TBI may increase risk for TBI diagnosis in the population with a negative head CT scan when another, non-TBI related condition could explain their clinical presentation (e.g. acute stress response).^30^ Diagnosis of TBI in the emergency department is complicated in patients with a negative head CT scan, as additional objective assessments for TBI have not been available until recently. As such, diagnosis of TBI in this population is largely based upon reported mechanism of injury, clinical signs (i.e. loss of consciousness, post-traumatic amnesia or alteration of mental status), and/or clinical symptoms.

In April 2024, the U.S. Food and Drug Administration approved the use of a whole blood test to measure peripheral GFAP and ubiquitin C-terminal hydrolase-L1 in patients with suspected TBI to determine if a CT scan is necessary. Increased utilization of these tests in the emergency department could help facilitate clinical decision-making for TBI diagnosis in the patient population with negative head CT scan, as prior work has suggested that ∼27% of patients with a negative head CT scan have a positive magnetic resonance imaging finding.^20^ GFAP and ubiquitin C-terminal hydrolase-L1 values are higher in the population with a negative head CT scan and positive magnetic resonance imaging compared with patients with a negative head CT and negative magnetic resonance imaging.^20^ Future research is critical to determine whether and how this test could be used as an aid-in-diagnosis of TBI among CT-negative individuals who present with head trauma. Such a test would reduce uncertainty for TBI diagnosis in this population and improve specificity of research studies like the present analysis. In other words, day-of-injury GFAP may have strong utility as a diagnostic biomarker of TBI but studies to date have shown limited utility of day-of-injury GFAP as a prognostic biomarker.^8^ Regardless, the present analysis suggests that, even in patients evaluated for TBI in the emergency department with negative head CT scans and lower GFAP, depressive disorder onset (or worsening) can occur and all patients should be monitored for these sequelae.

There was no association between day-of-injury GFAP and SI in this analysis. SI is a construct that can be consistent or transient and is largely dependent on an individual's current circumstances,^31,32^ which may or may not be related to their TBI. In other words, reporting current SI within the previous month could be related to biological changes in the brain or could be related to other experiential factors, such as unexpected life events.^33^ Major depressive disorder is more likely to be present over a longer time period and, as such, be associated with biological changes that may be detectable (such as lower GFAP levels).^27^ SI was not common in this cohort, with rates ranging from ∼4 to 5% depending on the follow-up timepoint. This also could have resulted in an inability to detect a real difference in GFAP between those with SI and those without due to small sample sizes.

### Limitations

The results of this study should be considered alongside several important limitations. The results of this study are only generalizable to adult patients who sought acute care for possible TBI at a level 1 trauma centre emergency department in the United States. This is a retrospective analysis of prospectively collected data, and the original TRACK-TBI was not statistically powered to analyse the relationship between blood biomarkers and mental health sequelae. The cohort had a high rate of positive CT scans at presentation, indicating the cohort may represent more severe TBIs with presenting GCS 13–15 than other cohorts with ‘milder’ TBI. This result may have occurred because enroling sites were level 1 trauma centres. History of psychiatric disorder and prior TBI were self-reported by the participant, which increases risk for possible recall bias. History of seizure was not explicitly documented at enrolment. Loss to follow-up was observed at typical rates compared with the broader TBI literature, but may nonetheless lead to biases in data interpretation.^34^ Depression status was determined based upon validated cut-offs from a depression screening tool, but may differ from what would be determined by an expert clinical interviewer. Other factors which could have potentially impacted study outcomes were not available for the current analysis, such as pain level.^22,35^ Other variables to consider in future research could be TBI history (of all severities) and number of prior TBIs, seeking and adherence to post-traumatic psychiatric treatment, substance abuse disorders, polytrauma, TBI symptoms, depression symptoms at time of injury, family history of depression or SI, attention deficit hyperactivity disorder history and years of education. These factors should be considered in future research. Subgroup analyses, especially for the SI comparisons, may be underpowered due to small sample sizes. Psychiatric sequelae 2 weeks post-injury can be quite different from the presence of psychiatric sequelae at 1-year post-injury. Future work with larger incidences of depression should consider these differences.

## Conclusion

The presence of intracranial abnormalities appeared to modify the relationship between day-of-injury GFAP levels and mental health sequelae after TBI with presenting GCS 13–15. Peripheral GFAP was inversely associated with odds of depression (but not SI) within the first year of TBI with negative head CT scan. Day-of-injury GFAP did not have a relationship to incident depression or SI in the first year post-injury amongst patients with evidence of acute intracranial trauma at presentation (i.e. CT+patients). Future work is needed to validate these findings, explore a potential mechanism for why lower biomarkers of acute brain trauma are associated with post-injury mental health issues and quantify any potential role of other blood biomarkers to prognostic models of post-injury depression or SI.

## Data Availability

De-identified data are available after submission of a data use agreement to the TRACK-TBI Executive Committee or by request from the Federal Interagency TBI Research Informatics System.^36^

## Appendix

**The TRACK-TBI Investigators:** Ann-Christine Duhaime, Adam R. Ferguson, Shankar Gopinath, Ramesh Grandhi, C. Dirk Keene, Christine Mac Donald, Amy Markowitz, Randall Merchant, Pratik Mukherjee, Laura B. Ngwenya, Claudia Robertson, Andrea Schneider, David Schnyer, Sabrina R. Taylor, Kevin Wang and Ross Zafonte
